# Prosystemin Overexpression in Tomato Enhances Resistance to Different Biotic Stresses by Activating Genes of Multiple Signaling Pathways

**DOI:** 10.1007/s11105-014-0834-x

**Published:** 2014-11-25

**Authors:** Mariangela Coppola, Giandomenico Corrado, Valentina Coppola, Pasquale Cascone, Rosanna Martinelli, Maria Cristina Digilio, Francesco Pennacchio, Rosa Rao

**Affiliations:** 1 Dipartimento di Agraria, Università degli Studi di Napoli Federico II, 80055 Portici, NA Italy; 2 CEINGE, 80145 Naples, Italy; 3 Institute for Plant Protection (CNR), 80055 Portici, NA Italy

**Keywords:** Systemin, *Solanum lycopersicum*, Resistance, Defense, DAMP

## Abstract

Systemin is a signal peptide that promotes the response to wounding and herbivore attack in tomato. This 18-amino acid peptide is released from a larger precursor, prosystemin. To study the role of systemin as a modulator of defense signaling, we generated tomato (*Solanum lycopersicum*) transgenic plants that overexpress the prosystemin cDNA. We carried out a transcriptomic analysis comparing two different transgenic events with the untransformed control. The Gene Ontology categories of the 503 differentially expressed genes indicated that several biological functions were affected. Systemin promotes the expression of an array of defense genes that are dependent on different signaling pathways and it downregulates genes connected with carbon fixation and carbohydrate metabolism. These alterations present a degree of overlap with the response programs that are classically associated to pathogen defense or abiotic stress protection, implying that end products of the systemin signaling pathway may be more diverse than expected. We show also that the observed transcriptional modifications have a relevant functional outcome, since transgenic lines were more resistant against very different biotic stressors such as aphids (*Macrosiphum euphorbiae*), phytopathogenic fungi (*Botrytis cinerea* and *Alternaria alternata*) and phytophagous larvae (*Spodoptera littoralis*). Our work demonstrated that in tomato the modulation of a single gene is sufficient to provide a wide resistance against stress by boosting endogenous defense pathways. Overall, the data provided evidence that the systemin peptide might serve as DAMP signal in tomato, acting as a broad indicator of tissue integrity.

**Electronic supplementary material:**

The online version of this article (doi:10.1007/s11105-014-0834-x) contains supplementary material, which is available to authorized users.

## Introduction

Plants have evolved a variety of defense mechanisms against biotic stress, including chemical compounds (e.g., toxins and anti-nutrient proteins), physical barriers (e.g., waxes, thorns, and trichomes), and indirect defenses that entail the recruitment of predators and parasitoids (Wu and Baldwin 2010). Many defenses are inducible, activated following the perception of herbivores or pathogens (Kessler and Baldwin 2002). In plants, a composite signaling cascade transcriptionally controls the production of a variety of chemically diverse metabolites involved in plant defense. Many induced responses are mediated by the interplay of relatively few phytohormones (Pieterse et al. 2012). There is a general agreement that jasmonic acid (JA), salicylic acid (SA), and ethylene (Et) play a dominant role in defense in nearly all plant species (Pieterse et al. 2012; Robert-Seilaniantz et al. 2011).

Insects, as well as other agents of biotic stress, induce also a defense reaction in undamaged distal tissues (systemically) (Heil and Bostock 2002; Kessler and Baldwin 2002; Wu and Baldwin 2010). This response implies the presence of signals that move through vascular tissues. Systemic signals can be different but are also related to jasmonates and salicylates (Farmer and Ryan 1990; Holopainen and Blande 2012). Moreover, volatile hormones (e.g., ethylene) and their derivatives (e.g., methyl jasmonate and methyl salicylate) participate in the systemic response to insect herbivores (Farmer and Ryan 1990; Holopainen and Blande 2012).

Systemin was the first identified plant bioactive peptide (Pearce et al. 1991). It was isolated from tomato as a potent inducer of protease inhibitors in both local and systemic leaves (Pearce et al. 1991). This 18 amino acids (aa) peptide is released from the C-terminal region of a larger precursor of 200 aa, called prosystemin (Mcgurl et al. 1992). Genetic evidences indicated that systemin most likely generates and amplifies a systemic signal, supporting the JA pathway (Lee and Howe 2003; Li et al. 2002; Schilmiller and Howe 2005). Upon wounding, systemin initiates a cascade that leads to the induction of early signaling components of the defense pathway and the resulting accumulation of molecules directly and indirectly affecting pests (Corrado et al. 2007; Mcgurl et al. 1994; Ryan 2000). At the cellular level, systemin induces a depolarization of the plasma membrane, the induction of mitogen-activated protein kinases (MAPKs), an increase of intracellular Ca^2+^ concentration and the activation of a phospholipase A_2_ (PLA2) (Ryan 2000; Sun et al. 2011). The latter is involved in the release of linolenic acid from membrane phospholipids, which primes the octadecanoid pathway and the downstream biosynthesis of 12-oxophytodienoic acid (OPDA) and jasmonic acid (Ryan 2000; Sun et al. 2011).

The role of systemin in the wounding or herbivore response was elucidated in a number of studies exploiting gain- or loss-of-function mutants. The overexpression of prosystemin induced the accumulation of protease inhibitors (PIs) that degrade essential amino acids in the herbivore midgut (Chen et al. 2005; Mcgurl et al. 1994). The silencing of prosystemin gene strongly reduces the plant response to herbivores (Orozcocardenas et al. 1993). More recently, it has been demonstrated that systemin plays a wider and more complex role. Tomato plants overexpressing prosystemin produced more volatile organic compounds (VOCs) (Corrado et al. 2007; Degenhardt et al. 2010) and were more attractive for parasitoids (Corrado et al. 2007). Furthermore, systemin is involved in tomato resistance against necrotrophic phytopathogens (Diaz et al. 2002; El Oirdi et al. 2011). Prosystemin overexpression also increased resistance to saline stress (Orsini et al. 2010).

All these features are usually discussed taking into account the overlap between different stress-related pathways. It has been demonstrated that the SA- and JA-pathways represent a flexible signaling network in plants (Koornneef and Pieterse 2008; Kunkel and Brooks 2002; Mur et al. 2006), which allows to fine tune the response against invaders and to minimize the fitness and metabolic cost associated to the defense reaction (Robert-Seilaniantz et al. 2011). As the different signaling pathways involved in stress response rely on modules composed of negative and positive regulatory components (Robert-Seilaniantz et al. 2011), it is likely that their balanced crosstalk is important to achieve a specific plant response to biotic stress. This is also demonstrated by the fact that the modification of the hormonal crosstalk in attacked plants seems to be a common strategy for different biotic stress (El Oirdi et al. 2011; Robert-Seilaniantz et al. 2011; Walling 2000).

Recent progress has revealed that biotic stress can generate larger than expected sets of differentially expressed genes, which usually include a significant proportion of sequences that do not code for proteins directly involved in plant defense (Bilgin et al. 2010; Coppola et al. 2013; De Vos et al. 2005; Kerchev et al. 2012; Thompson and Goggin 2006). These works also indicated that the interactions between signaling pathways are complex and not yet fully discovered. However, whether or not connected transmission processes that originate from a single signaling molecule may cooperate to influence significantly various pathological outcomes has not been frequently addressed.

Signaling peptides like systemin are believed to be crucial in the control of important plant functions by modulating different response pathways. With the aim of understating the molecular mechanisms that trigger the tomato response to phytophagous pests, we analyzed the transcriptomic changes associated to the overexpression of prosystemin and evaluated its effect against different damaging organisms. Our data led to a more comprehensive understanding of the systemin defense network, highlighted that the positive and negative regulation of the expression of genes involved in diverse molecular pathways has an extensive functional outcome in the tomato-biotic stress interactions, and indicated that the systemin peptide has the ability to reinforce responses towards different biotic stresses.

## Materials and Methods

### Tomato Genetic Transformation

Considering that the hybrid tomato cultivar “Better Boy” that was originally transformed with a construct overexpressing prosystemin cDNA (Mcgurl et al. 1994) is a VFN variety (i.e., resistant to Verticillium wilt, Fusarium wilt, root-knot nematodes and aphids), we used as a recipient a susceptible genotype, the “Red Setter” variety, more appropriate to study systemin effect on biotic stresses. Furthermore, since this variety is a non-hybrid cultivar, it is also possible to have genetically uniform progenies. Seeds of *Solanum lycopersicum* L. “Red Setter” were sterilized and germinated in vitro on TRI1 medium (2.2 g l^−1^ MS salts, 0.2 mg l^−1^ thiamine, 50 mg l^−1^ myo-Inositol, 0.2 mg l^−1^ IAA, 15 g l^−1^ sucrose; pH 5.9) solidified with 8 g l^−1^ agar (Duchefa). *Agrobacterium tumefaciens* (strain C5851) containing the pMZ plasmid (Rocco et al. 2008) was grown in AB medium (60 g l^−1^ K_2_HPO_4_, 20 g l^−1^ NaH_2_PO_4_, 20 g l^−1^ NH_4_Cl, 3 g l^−1^ KCl, 5 g l^−1^ glucose, 6 g l^−1^ MgSO_4_·7H_2_O, 0.2 g l^−1^ CaCl_2_, 50 mg l^−1^ FeSO_4_·7H_2_O) supplemented with 500 mg l^−1^ streptomycin and 50 mg l^−1^ kanamycin. *A. tumefaciens*-mediated transformation of tomato cotyledons was performed as described (Vanroekel et al. 1993) with a different co-cultivation time (2 days) and without using a feeder layer. Putative transformants were selected on kanamycin (50 mg l^−1^) and transferred to sterile soil in an environmental chamber 26 + 2 °C with a photoperiod of 16:8 h light/dark. Homozygous T2 populations from two independent single-copy transgenic events were selected for molecular analysis and bioassays.

### Molecular Analysis of Plants

DNA was isolated from leaves of 2-week-old plants as described (Fulton et al. 1995). For Southern blot analysis, total DNA (5 μg) was digested with *Hin*d III (Promega), resolved into a 0.7 % (*w*/*v*) agarose gel and transferred to a Hybond-N membrane (Amersham) and cross-linked to the membrane by UV (120 mJ) as reported (Corrado et al. 2005). A DIG-labeled probe was obtained using Sys Fw and Sys Rv primers (Supplementary Table 1). Filters were pre-hybridized, incubated with 500 ng of a DIG-labeled probe and washed according to the instruction of the DIG-High Prime DNA Labeling and Detection Starter Kit (Roche). Probe-target hybrids were detected with an enzyme-linked chemiluminescent immunoassay using the CDP-Star substrate (Roche).

The isolation of total RNA from leaves of 4-week-old plants, the synthesis of the first strand cDNA and real-time PCRs were performed as already reported (Corrado et al. 2012). Gene expression analysis was carried out using two technical replicates for each of the three biological replicates per samples. Relative quantification of gene expression was carried out using the 2^−ΔΔCt^ method (Livak and Schmittgen 2001). The statistical significance was evaluated using the Student’s *t* test. The housekeeping gene EF-1α was used as an endogenous reference gene for the normalization of the expression levels of the target genes. Primers and their main features are reported in the Supplementary Table 1.

For western blot analysis, total soluble proteins were isolated from leaves. Briefly, 0.5 g of leaf tissue were finely grounded in liquid nitrogen and suspended in 300 μl of extraction buffer (6 M Urea, 50 mM Tris–HCl pH 7.5, 50 mM NaCl, 5 mM EDTA; pH 8.0). Proteins were resolved by SDS-PAGE using a 6 % (*w*/*v*) stacking-12 % (*w*/*v*) separation polyacrylamide gel in a Mini-Protein II (Bio-Rad) apparatus, and transferred onto nitrocellulose membrane by electroblotting with a Mini Trans-Blot Cell system (Bio-Rad). The membrane was probed with anti-prosystemin polyclonal antibody (Narváez-Vásquez and Ryan 2004) (dilution 1:1000), and anti-rabbit IgGs conjugated with peroxidase (Santa Cruz Biotechnology; dilution 1:2500) as described (Tortiglione et al. 2003). Detected proteins were visualized using a chemiluminescent detection system (ECL, GE Healthcare) using Hyperfilm ECL (GE Healthcare). Molecular weights were estimated by comparison with the PageRuler Plus Prestained Protein Ladder (Fermentas).

### Two-Color Microarray-Based Gene Expression Analysis

We used three biological replicates per genotype. For each replicate, three leaves of a 4-week-old plant were pooled to reduce noise arising from biological variation. Leaf tissue was powdered in liquid nitrogen and homogenized in Qiazol (Qiagen). Total RNA was extracted using Plant RNeasy mini kit (Qiagen) according to the manufacturer’s protocol. Samples were analyzed with the 2100 Bioanalyzer system (Agilent Technologies) for sizing, quantitation, and quality control of RNA. Only samples with a 260/280 nm absorbance >1.8 and a 260/230 nm absorbance >2 were amplified in the presence of cyanine-3- or cyanine-5-labeled CTP using the Agilent Low Input Quick Amp Labeling kit (Agilent Technologies). Samples were purified using the RNeasy mini spin columns (Qiagen). The quality of labeled targets was determined by calculating the amount of cDNA produced, the picomoles of dye incorporated and the frequency of incorporation, with a NanoDrop 1000 (Thermo Scientific). Equal amounts of cRNAs (825 ng) from control and a transgenic line were mixed together and hybridized to the Tomato Gene Expression Microarray 4x44K (Agilent Technologies) at 65 °C for 17 h in an Agilent Hybridization Oven (G2545A) at 10 rpm. Slides were washed with the Gene Expression Wash buffer 1 for 1 min at room temperature, the Gene Expression Wash buffer 2 for 1 min at 37 °C, and treated with the Stabilization and Drying Solution for 30 s at room temperature. Slides were scanned with a dual-laser microarray scanner (G2565AA, Agilent Technologies) and image data were processed using the Feature Extraction v. 10 software (Agilent Technologies). Raw data and associated sample information were processed by Genespring GX 10 (Agilent Technologies). Statistical analysis was performed using background-corrected mean signal intensities from each dye channel. Microarray data were normalized using intensity-dependent global normalization (LOWESS). Differentially expressed RNAs were identified using a filtering by the Benjamini and Hochberg False Discovery Rate (*p* < 0.05) and a minimum of a two-fold variation in expression compared to untransformed controls.

### Functional Annotation

The sequence of each differentially expressed probe was downloaded from NCBI starting from the available information provided by the microarray manufacturer (Agilent Technologies). Functional annotation was carried out by sequence analysis using the Blast2GO software (Gotz et al. 2008). Briefly, a BlastX similarity search against the nr NCBI protein database was performed to retrieve a maximum of 20 homologous hits per query. GO-term mapping and annotation were retrieved using NCBI as well as non-redundant reference protein database (PSD, UniProt, Swiss-Prot, TrEMBL, RefSeq, GenPept, and PDB Full Gene Ontology DB). Additional annotations (e.g., the recovery of implicit “Biological Process” and “Cellular Component” GO terms from “Molecular Function” annotations) were implemented using ANNEX. Completion of the functional annotation with protein domain information was obtained with InterProScan 5.0. Mapping of enzymatic activities into molecular pathways was acquired from the KEGG database.

### *Lepidoptera* Bioassay

*Spodoptera littoralis* larvae were grown in an environmental chamber at 25 ± 2 °C, 70 ± 5 % RH and fed with an artificial diet composed by 41.4 g l^−1^ wheat germ, 59.2 g l^−1^ brewer’s yeast and 165 g l^−1^ corn meal, supplemented with 5.9 g l^−1^ ascorbic acid, 1.8 g l^−1^ methyl 4-hydroxybenzoate and 29.6 g l^−1^ agar. About 60 eggs were hatched on this artificial diet and allowed to grow until the second instar. Uniform second instar larvae, were selected and separated in three groups of 12–15 members and each group was used to evaluate larval weight and survival rate after feeding on control, RSYS 24 and RSYS32 leaf disks. Single larvae were isolated in a tray well (Bio-Ba-8, Color-Dec, Italy) covered by perforated plastic lids (Bio-Cv-1, Color-Dec Italy), containing 2 % agar (*w*/*v*) to create a moist environment required to keep turgid the experimental tomato leaf disks. Larvae were daily offered leaf disks of uniform size, initially of 2 cm^2^, later of 3, 4, and 5 cm^2^ following larvae growth.

Plastic trays were kept at 28 °C 16:8 h light/dark photoperiod. Larval weight and mortality were recorded until pupae development. Fourth instar larvae were transferred into plastic boxes containing vermiculite for pupae development. Data were collected from two experimental replications.

### Necrotrophic Fungi Bioassay

Five-week-old plants were tested for resistance to *Botrytis cinerea* and *Alternaria solani* as previously described (Corrado et al. 2005). Briefly, spores of *B. cinerea* and *A. solani* were suspended in sterile distilled water, filtered through sterile Kimwipes (Kimberly-Clark) to remove fragments of hyphae and adjusted to a concentration of 1 · 10^6^ conidia per ml. Ten μl of the spore suspension were applied between the leaf veins, using four different inoculation points per leaf. The assay with detached leaves was carried out using four plants per genotype. For each plant, two leaves were placed on sponges soaked in sterile water and incubated in a growth chamber at 23 °C, under 16:8 h light/dark photoperiod and 90 % RH. The size of the lesions was measured after 48, 72, and 96 h. For the whole plant assay, inocula were performed on four plants per genotype, and lesions were measured after 48 and 96 h. Lesion dimensions were measured using a digital caliber (Neiko 01407A).

### Aphid Bioassays

A clonal culture of *Macrosiphum euphorbiae* was reared on *S. lycopersicum* cv. “San Marzano” in an environmental chamber at 20 ± 2 °C, 65 ± 5 % RH and a 16:8 h light/dark photoperiod. For bioassays, 4-week-old plants were placed inside wood frame cages covered with mesh and infested with synchronized 1-day-old nymphs of *M. euphorbiae*. Assays were carried out at 20 ± 2 °C, 65 ± 5 % RH, 16:8 h light/dark photoperiod using in total 34 Red Setter and 52 RSYS24 plants. Aphids were free to move on the same plant or to reject the host by dropping off. As new-born nymphs are very sessile during their first days of life (Klingauf 1987), the number of nymphs present on each plant after 24 h was used as an estimate of adult preference (Poch et al. 1998). Mortality was calculated counting the number of surviving aphids after 48 h. Values were normalized using the Henderson-Tilton formula (Dent 2000). Subsequently, the number of aphids and the presence of exuviae were monitored daily for 10 days.

For the weight increase assay, 20 adult apterous aphids were placed on three plants per genotype. Aphids were weighted before being transferred to the host plant and after 48 h of feeding. The statistical significance of the weight increase was assessed by a Student’s *t* test. The weight of the aphids was not statistically different between experimental groups at the beginning of the test (*p* < 0.01).

## Results

### Generation and Analysis of the RSYS Transgenic Lines

**Fig. 1:**
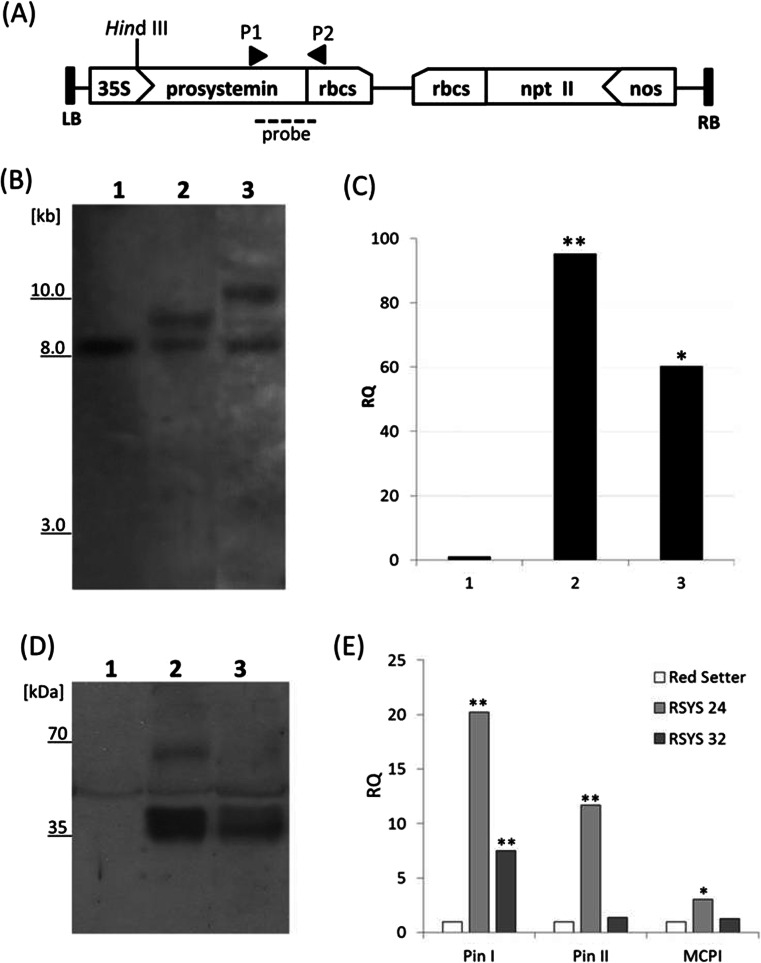
Molecular analysis of the transgenic lines. **a** T-DNA map of the pMZ binary vector used for plant genetic transformation showing the restriction site used for Southern blot analysis (not to scale). *Arrows* represent promoter sequences, and *black triangles* indicate the location of the primers P1 (Sys Fw) and P2 (Sys Rv) used for probe synthesis and RT-PCR. A *dashed line* underlines the region hybridized in the Southern analysis. *LB* T-DNA left border sequence; *35S* CaMV35S RNA gene promoter; *prosystemin* prosystemin cDNA sequence; *rbcS* poly(A) addition sequence of the pea rbcS; *nptII* neomycin phosphotransferase II coding sequence; *nos* nopaline synthase promoter; *RB* T-DNA right border sequence. **b** Southern blot analysis. *1* Red Setter, *2* RSYS 24, *3* RSYS 32. *Numbers at the left margin* indicate marker fragment sizes in kilobase pairs. **c** Relative quantification (*RQ*) of the prosystemin expression by real-time RT-PCR. *1* Red Setter, *2* RSYS 24, *3* RSYS 32. Quantities (RQ) are shown relative to the calibrator genotype Red Setter. Data statistical significance was calculated using Student’s *t* test (*p < 0.05; **p < 0.01). **d** Western blot analysis. *1* Red Setter, *2* RSYS 24, *3* RSYS 32. *Numbers at the left margin* indicate marker sizes in kilodaltons. **e** Relative quantification (*RQ*) of the expression of proteinase inhibitors by real-time RT-PCR. *Pin I* proteinase inhibitor I, *Pin II* proteinase inhibitor II, *MCPI* metallocarboxypeptidase proteinase inhibitors. Quantities (RQ) are shown relative to the calibrator genotype Red Setter. Data statistical significance was calculated using Student’s *t* test (*p < 0.05; **p < 0.01)

Tomato plants (*S. lycopersicum* L.) were stably transformed via Agrobacterium with a construct containing prosystemin cDNA under the control of the Cauliflower Mosaic Virus 35S RNA promoter. A schematic representation of the transgenic T-DNA is shown in Fig. 1a. Putative transformants, named RSYS, were screened by PCR (not shown) and Southern blot hybridization to confirm the presence and the number of T-DNA insertions (Fig. 1b). Transgene expression was analyzed by using a real-time RT-PCR approach. Figure 1c reports the quantification of prosystemin expression relative to the untransformed control. The accumulation of the prosystemin pro-hormone in leaves was monitored by Western blot assay (Fig. 1d). A protein with an apparent molecular weight of around 37 kDa was detected in the transgenic lines. The apparent mass of the prosystemin protein does not correspond to the predicted mass (23 kDa), most likely because of the high percentage (44 %) of charged amino acids (Delano et al. 1999). Among the 13 transformants that overexpressed prosystemin cDNA, two lines, namely RSYS24 and RSYS32, were selected, as they show a high level of expression and have a single transgenic locus. The phenotype of these transgenic lines is presented in the Supplementary Figure 1. As prosystemin overexpression generates a signal that constitutively induces proteinase inhibitors synthesis (Mcgurl et al. 1994), we also evaluated the expression level of three genes coding for proteinase inhibitors (proteinase inhibitor I, II and metallocarboxypeptidase proteinase inhibitor) by real-time RT-PCR. The three genes were upregulated in the transgenic plants (Fig. 1e), indicating the generation of a signal that induces constitutive proteinase inhibitor synthesis.

### Functional Annotation of the Genes Activated by Prosystemin Overexpression

The transcriptomic changes imposed by the constitutive prosystemin overexpression were monitored by using the Tomato Gene Expression 4x44k array (Agilent). A comparative gene expression analysis was performed with cDNAs from leaves of the two selected transgenic genotypes (RSYS24 and RSYS32) and Red Setter untransformed controls. After filtering, differentially expressed transcripts were identified using a fold-change cut-off ≥2.0 and a *p* < 0.05 (Benjamini and Hochberg False Discovery Rate). This analysis identified 689 differentially expressed probes that, according to the available reference tomato genome, correspond to 503 genes. Approximately, three quarters (74 %) were upregulated in the transgenic lines, while the remaining downregulated (Supplementary Tables 2 and 3, respectively). To validate microarray results, the expression of ten differentially expressed genes was analyzed by real-time PCR. The Supplementary Figure 2 shows the concordance between microarray log two-fold change and log two real-time RQ values, on a linear scale. The results indicated a significant (*p* < 0.01) and high correlation (0.96; Pearson coefficient) between the two data sets.

Functional annotation of the differentially expressed sequences and data mining on the resulting annotations was based predominantly on the gene ontology (GO) vocabulary. For the “biological process” domain, a broader overview of the ontology content was achieved by using the GO-plant slim list, to limit the detail of the specific fine-grained terms.

**Fig. 2:**
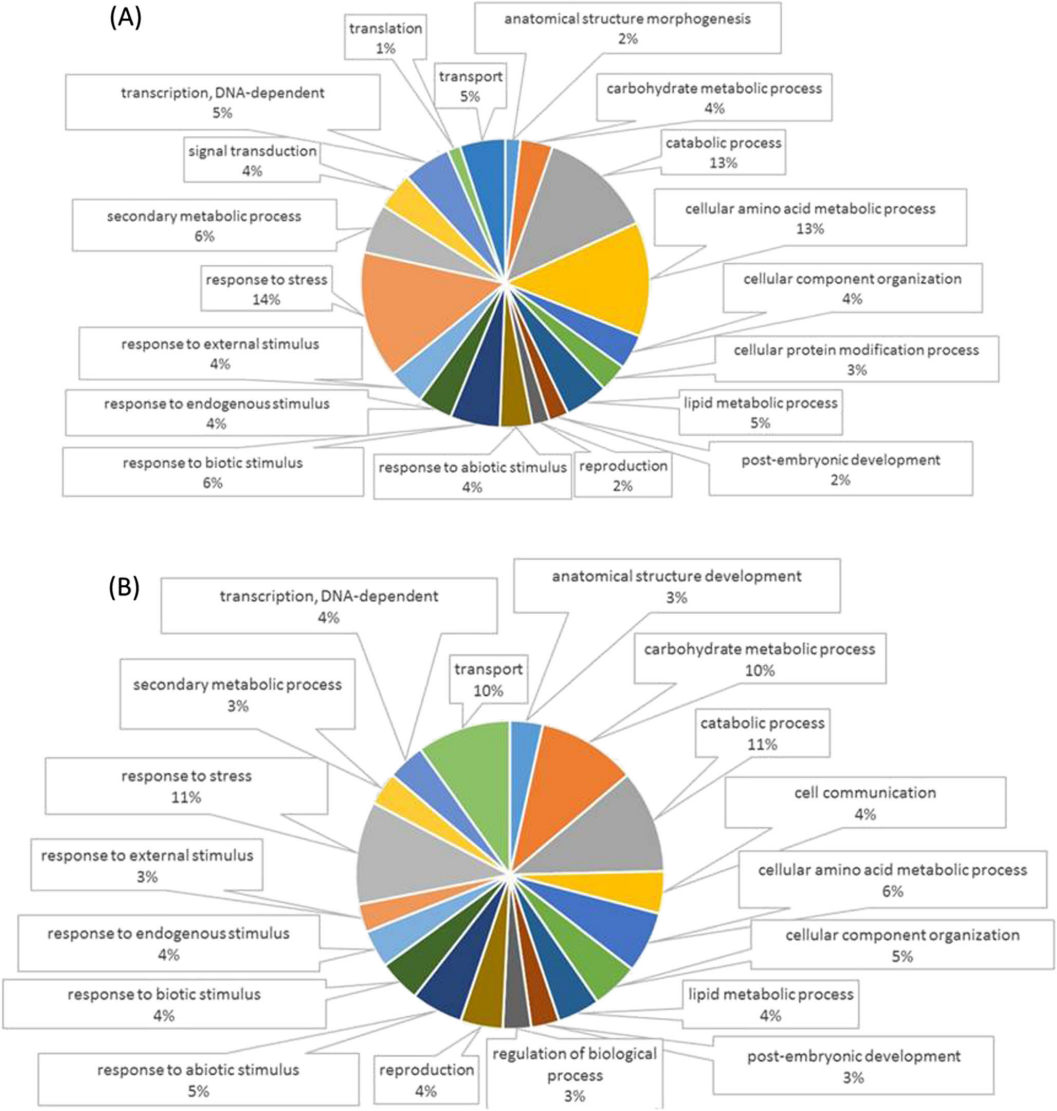
Multilevel pie charts representation of GO-annotation results. Relative distribution of “Biological Process” terms following GO classification of the upregulated (**a**) and downregulated (**b**) genes (sequence cut-off > 5). A multilevel pie was obtained using the lowest GO terms per branch that fulfill the annotation weight criteria (sequence abundance; cut-off > 5)

Considering the “biological process” GO terms, the multilevel distribution of the upregulated sequences indicated that “response to stress”, “cellular amino acid metabolic process”, and “catabolic process” were the most relevant series of molecular events or functions affected by prosystemin overexpression (Fig. 2a; Supplementary Table 4). In quantitative terms, other relevant processes were “response to biotic stimulus”, “secondary metabolic process”, “transcription, DNA-dependent”, and “transport”. For the downregulated sequences, the most represented GO terms were “catabolic process”, “response to stress”, “carbohydrate metabolic process”, and “transport” (Fig. 2b; Supplementary Table 5).

The categorization of the annotated genes using the KEGG database indicated differences between the metabolic pathways affected by the up- and the downregulated genes (Supplementary Tables 6 and 7, respectively). According to the absolute number of sequences involved, prosystemin mainly increased the metabolism of several amino acids (cysteine, methionin, arginine, proline, tyrosine, glycine, serine, and trypthophan), of the alpha-linolenic and fatty-acid pathways, and of phenylpropanoids-related pathways. Prosystemin downregulated genes whose enzymatic activities were often included in carbohydrate metabolism, such as starch, sucrose, galactose, fructose, mannose, amino sugar, and nucleotide sugar (i.e., involved in glycosylation) metabolism, as well as carbon fixation.

### Effects of Prosystemin Overexpression on the Tomato Transcriptome

As expected, prosystemin increased the expression of genes involved in the biosynthesis of jasmonates (i.e., a chloroplastic 13*S*-lipoxigenase, a peroxisomal ketoacyl-thiolase, a phospholipase and an allele oxide synthase) and of a mono-oxygenase involved in the production of linoleic acid. Nonetheless, the Jasmonate Zim Domain (JAZ) protein 1 and 3 were also overexpressed. In *Arabidopsis*, JAZ proteins are considered negative regulators for JA-responsive genes (Chung et al. 2008). Degradation of JAZ repressors triggered by ubiquitin-proteasome induces the expression of transcription factors such as MYC2 driving the expression of JA-related genes in response to tissue injury or other stresses. Systemin-dependent activation of the tomato JAZ proteins 1 and 3 is most likely a consequence of the high level of the JA signaling, so it could represent a strategy of feedback control or energetic costs reduction. Besides their roles in regulating developmental processes, plant hormones are involved in signaling networks related to the stress response (Robert-Seilaniantz et al. 2011). Moreover, the transgenic lines overexpressed ethylene transcription factors (6) and two genes coding for amino acid hydrolases that release active IAA from conjugates. Although plausible, it is not experimentally known if these tomato enzymes also hydrolyze amino acid-conjugated forms of JA such as the jasmonoyl isoleucine, considered the bioactive JA derivative needed for long-distance signaling (Katsir et al. 2008). Prosystemin also upregulated a gene coding for a det2-like (steroid 5-alpha-reductase-like) protein and of a 3-beta-hydroxysteroid-delta-isomerase-like, enzymes involved in the early steps of brassinosteroid biosynthesis (Fujioka et al. 1997). Furthermore, SA responsive genes, such as those coding for different classes of pathogenesis-related proteins (i.e., osmotin, β-glucosidase 13, pathogenesis-related protein 10, pto-responsive gene 1 protein, defensin, endochitinase, and thaumatin) were upregulated. The data imply that different hormone signal transductions play a role in systemin-mediated responses. Accordingly, the effect of prosystemin overexpression involved also molecular sensors whose abundance is affected by a range of environmental cues such as Ca^2+^ and reactive oxygen species (ROS) (Kessler and Baldwin 2002; Wu and Baldwin 2010). Calcium-dependent kinases and calmodulins were overexpressed in the transgenic plants, as well as antioxidant enzymes (four glutathione *S*-transferase-like proteins; a thioredoxin m).

Twelve overexpressed sequences are connected with enzymatic activities associated to the phenylpropanoid pathway. In plants, the biosynthesis of all phenylpropanoids begins with the amino acids phenylalanine and tyrosine. In the transgenic lines, both phenylalanine ammonia-lyases (PAL), responsible for the transformation of l-phenylalanine into *trans*-cinnamic acid, and *trans*-cinnamate 4-monooxygenases/hydrolases (C4H), responsible for the transformation of *trans*-cinnamate into 4-hydroxycinnamate (*p*-coumaric acid), were overexpressed. Phenylpropanoid metabolism generates an array of secondary metabolites, including phenylpropanoid esters, flavonoids, and anthocyanins, which have been linked to plant signaling and defense against biotic or abiotic stress (Naoumkina et al. 2010; Vogt 2010). Moreover, the products derived from the dehydrogenative polymerization of the three monolignol precursors deriving from the PP are also essential for the biosynthesis of lignin. The biosynthetic flux of monolignols is controlled in different steps but, in addition to the phenylalanine supply, the elements that are considered important are the cinnamate 4-hydroxylase (C4H) and of *p*-coumarate 3-hydroxylase (CH_3_) activities (Anterola and Lewis 2002; Naoumkina et al. 2010). Prosystemin increased also the expression of a *p*-coumarate 3-hydroxylase, suggesting a variation of the strength and stiffness of the secondary cell wall. In addition, genes related to the flavonoid biosynthesis (e.g., leucoanthocyanidin dioxygenase-like, 2-oxoglutarate Fe-dependent dioxygenase-like, caffeoyl-*o*-methyltransferases, hydroxycinnamoyl transferases) were also upregulated in leaves. Besides their role in pigmentation of flowers, fruits pollen, and seeds, flavonoids play a role in plant adaptation to harsh environmental conditions, as well as in the interaction between plants and biotic stress. For instance, the amount and type of flavonoids are important determinants of the leaf taste and can strongly deter feeding by herbivores (Aron and Kennedy 2008; Dixon 2005; Harborne and Williams 2000).

The most abundant class of direct gene products involved in defense was represented by protease inhibitors (18 sequences), such as inhibitors of metallocarboxypeptidases, cysteine, trypsin, and kunitz-type proteases. Other overexpressed genes that can directly affect pest performance were eight polyphenol oxidases, three leucine aminopeptidases, two serine carboxypeptidases, two arginine decarboxylases, and one threonine deaminase. The data indicated that prosystemin can boost the synthesis of a range of proteins to impair digestive processes in the insect gut. Moreover, genes coding for proteinase inhibitors showed the highest fold change, representing, for instance, eight of the top ten highly expressed genes (Supplementary Table 2). Polyphenol oxidases represent the second highly expressed class of genes. Prosystemin also activated genes that may affect indirect defense against pests. The transcriptional analysis indicated the upregulation of gene-encoding enzymes of the terpenoid biosynthetic pathway (3-beta-hydroxysteroid-Delta (8), Delta (7)-isomerase-like, geraniol 8-hydroxylase-like, putative monoterpene synthase 1). Compounds produced by this pathway are major components of the Volatile Organic Compound blend (Holopainen and Blande 2012; Walling 2000).

Transgenic plants also overexpressed genes involved in abiotic stress tolerance such as two spermidine- and five spermine-synthases, which promote the formation of higher molecular weight polyamines (Alcazar et al. 2010). Other overexpressed genes were those coding for a desiccation responsive protein, a late embryogenesis-abundant protein, a DNA repair protein uvh3-like, and a annexin. Annexins are calcium-dependent phospholipid-binding proteins with a peroxidase activity, which often participate in plant response to stress (Gorecka et al. 2007).

Prosystemin also alters the expression of genes involved in primary metabolic processes and several genes involved in sugar metabolism and carbon fixation were downregulated. Specifically, seven genes involved in the amino sugar and nucleotide metabolism, seven in starch and sucrose metabolism, five in galactose metabolism, three in fructose and mannose metabolism and three genes in carbon fixation (including a ribulose bisphosphate carboxylase small chain) were expressed at lower level in the transgenic lines. The data imply that such reduction could be a genetically programmed plant response, rather than an effect of a direct interaction with an herbivore (e.g., water loss from damaged tissue), as widely reported (Berger et al. 2007; Ishiga et al. 2009; Nabity et al. 2013). Despite these evidences, recently Attaran and collaborators (2014) demonstrated that in *Arabidopsis* the downregulation of the expression of genes involved in photosynthesis following defense elicitation is not always correlated with the reduction of photosynthetic efficiency. The authors suggest that, in *Arabidopsis*, the photosynthetic system endures variation in the expression of components without significant reduction in quantum efficiency of photosystem II. Therefore, we cannot exclude that, although RSYS plants show a significant downregulation of transcripts of photosynthesis associated genes, their photosynthetic efficiency remain unaltered.

Transcriptional control of stress-responsive genes is a crucial part of the plant response and systemin also affected the expression of transcription factors (TF). In addition to the above-mentioned ERF genes, a member of the other main families of TFs related to stress (i.e., bZIP and WRKY proteins) were differentially expressed. Among others, TFs related to heat-stress were differentially expressed (two up- and one downregulated). A MYC-type ICE1-like TF that increases the amount of different osmolyte (Feng et al. 2013) was overexpressed, while a BIM1-like TF, a bHLH protein involved in brassinosteroid signaling, was downregulated.

### Evaluation of the Resistance Against Phytophagous Larvae

**Fig. 3:**
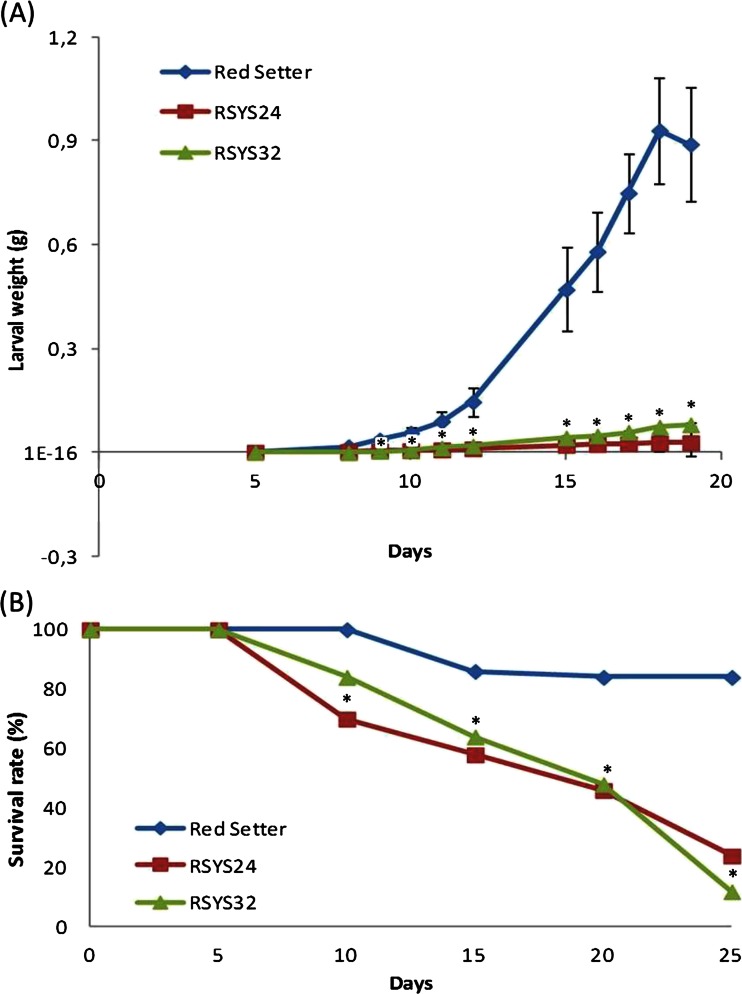
Effect of prosystemin overexpression on *Spodoptera littoralis* larvae. **a** Average weight (±s.d.) of *S. littoralis* larvae feed on transgenic or control leaves. At each time points, values were significantly different between controls and transgenic lines starting from 8 days (*p < 0.05; Student’s *t* test); **b** Survival rate of larvae feed on transgenic or control leaves

In order to assess in our transgenic lines the resistance conferred by prosystemin overexpression against *Lepidoptera* (McGurl et al. 1992), plants were assayed against the cotton leafworm (*S. littoralis*), a pest on vegetables, fruits, flowers, and other crops. After 8 days, the weight of the larvae fed with transgenic leaves started to be significantly lower (Fig. 3a). Larvae survival rate was also reduced during the whole bioassay (Fig. 3b). After 25 days of feeding, the survival rate was 24 % for RSYS24 and 12 % for RSYS32, compared to 84 % for the untransformed plants. The data indicated that prosystemin overexpression compromised severely both growth and survival of the *S. littoralis* larvae.

### Prosystemin Overexpression Increases Tomato Resistance Against Necrotrophic Fungi

**Fig. 4:**
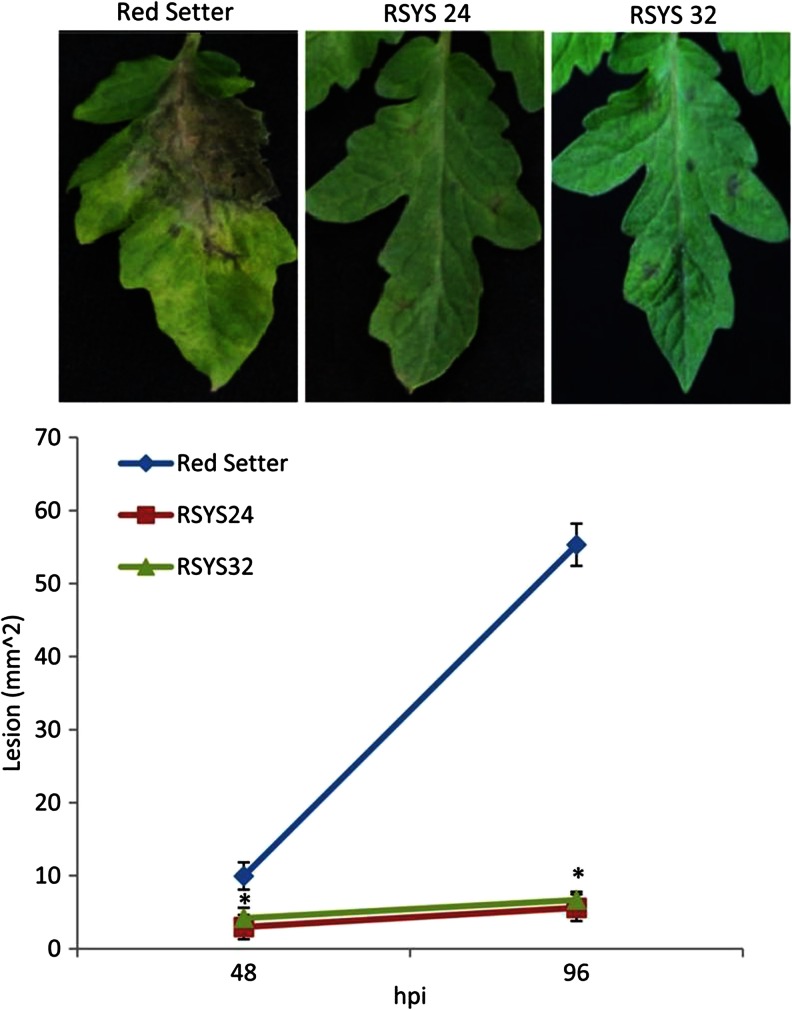
Effect of prosystemin overexpression on *Botrytis cinerea* in whole plant assay. Response to *B. cinerea* artificial infection by leaves from untransformed (*Red Setter*) and transformed lines (*RSYS 24* and *RSYS 32*). The graphs display the average (±s.d.) of the lesion size at 48, 96 h post infestation (hpi). At each time points, values were significantly different between controls and transgenic lines starting (*p < 0.05; Student’s *t* test)

**Fig. 5:**
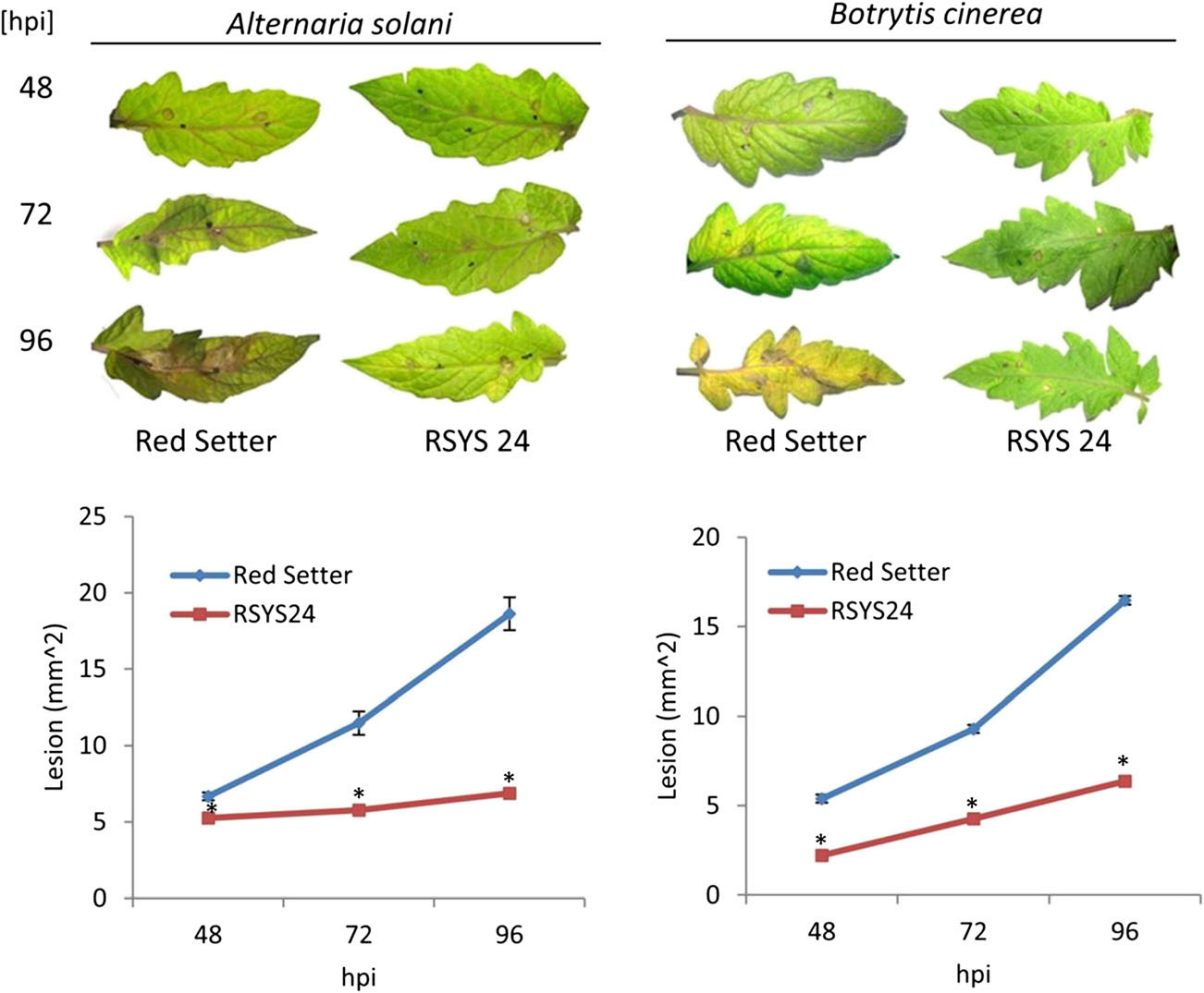
Effect of prosystemin overexpression on *Alternaria solani* and *Botrytis cinerea* in detached leaf assay. Response to *A. solani* and *B. cinerea* artificial infection bt untransformed (*Red Setter*) and transformed leaves (*RSYS 24*). The graphs display the average (±s.d.) of the lesion size at 48, 72, and 96 h post infestation (hpi). At each time points, values were significantly different between controls and transgenic lines starting (*p < 0.05; Student’s *t* test)

We observed that among overexpressed genes in RSYS plants there are transcripts induced by necrotrophic fungi and therefore possibly involved in the enhancement of inducible defenses. In addition, systemin was previously reported to be involved in plant tolerance to *B. cinerea* (Diaz et al. 2002; El Oirdi et al. 2011). Therefore, we evaluated the performance of 4-week-old RSYS plants to necrotrophic fungi. Two different experiments were carried out: one on whole plants and the other on detached leaves. Disease severity was quantified by measuring the necrotic areas. The results of the first experiment are shown in Fig. 4. Transgenic leaves of both RSYS24 and RSYS32 plants display a strong reduction of fungi induced lesions (Fig. 4). We then extended the evaluation of tolerance to *A. solani*, a necrotrophic pathogen known to attack tomato leaves. In this experiment, we used detached leaves of RSYS24 that were inoculated with *A. solani* or *B. cinerea* (Fig. 5). For both pathogens, lesions development was significantly reduced in prosystemin overexpressing leaves.

**Fig. 6:**
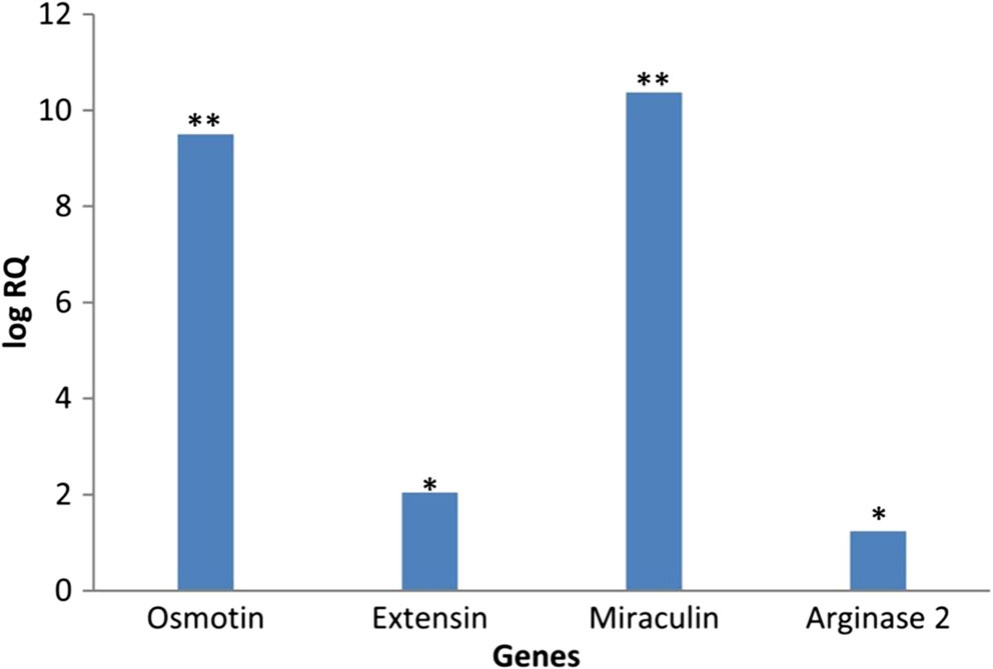
Expression analysis of selected genes in Red Setter leaves following *Botrytis cinerea* inoculation. Relative quantification (log RQ) of gene expression by real-time RT-PCR of selected differentially expressed genes. The 2^−ΔΔCt^ values were significantly different between treated and control plants (*p < 0.05; **p < 0.01; Student’s *t* test)

The resistance to necrotrophic fungi has been described to be also JA/Et-mediated (AbuQamar et al. 2008; Glazebrook 2005). Therefore, to get new insights on the role of prosystemin as enhancer of the resistance to fungal pathogens, we examined the expression levels of genes that are studied often in relation to plant-microbe interaction. From the differentially expressed genes, we evaluated the expression of *osmotin*, *extension*, and *miraculin* in leaves of Red Setter plants following *B. cinerea* infection. Furthermore, we also studied the expression of the Arginase 2. Arginases are enzymes involved in the biosynthesis of polyamines that accumulate in plants in response to environmental stimuli (Chen et al. 2004). *B. cinerea* infestation of ‘Red Setter’ plants upregulates the selected prosystemin-activated genes, implying that the resistance of the transgenic plants is likely to be dependent by the constitutive expression of an array of different genes (Fig. 6).

### Prosystemin Overexpression Increases Aphid Resistance in Tomato

To evaluate if the constitutive expression of prosystemin increases resistance against phloem-feeders, plants of the RSYS 24 line were assayed against *M. euphorbiae*. The results of the no-choice test experiments indicated that *M. euphorbiae* did not avoid transgenic plants compared to the untransformed control (Table 1). On the other hand, both longevity and mortality were different between the genotypes under investigation. An increased tolerance of the transgenic plants was also indicated by the lower adults weight increase. Although aphids tend to colonize the transgenic plants when no other choice is offered, the data indicated that prosystemin overexpression enhances aphid antibiosis in tomato.

**Table 1:** Effects of the prosystemin overexpression on *Macrosiphum euphorbiae*

Host genotype	Non acceptance (significance)^a^	Corrected mortality^b^	Longevity decrease^c^	Weight increase in mg (significance)^d^
Red Setter	25.9 %	–	–	11.6
RSYS 24	23.8 % (n.s.)	45.2 %	39.9 %	4.3 (*p* < 0.01)

^a^The significance was estimated by a chi-square test on raw data (*n.s*. not significant, *p* > 0.05)

^b^Mortality data were taken 48 h following infestation and normalized using the Henderson-Tilton adjustment

^c^Longevity was measured on a daily base considering only the aphids that accepted the host plant

^d^After 48 h, the weight was measured on the remaining 17 aphids for the Red Setter genotype and 14 aphids for the RSYS 24 genotype. The significance was estimated by the Student *t* test

**Fig. 7:**
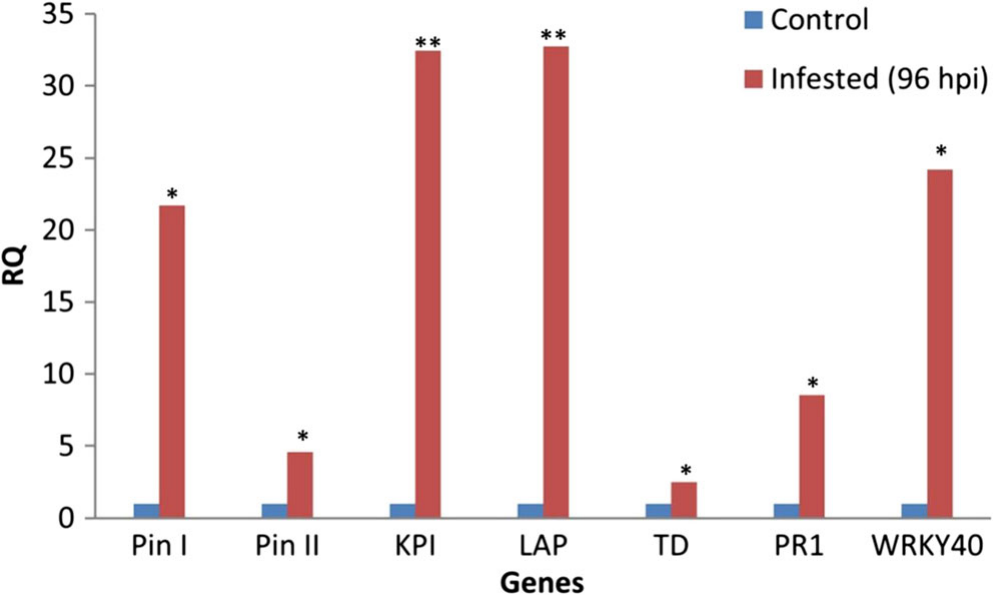
Expression analysis of selected genes in *Red Setter* leaves following aphid infestation. Relative quantification (RQ) of gene expression by real-time RT-PCR of selected differentially expressed genes. *Pin I* proteinase inhibitor I, *Pin II* proteinase inhibitor II, *KPI* Kunitz-type proteinase inhibitor family protein, *LAP* leucine amino peptidase, *TD* threonine deaminase, *PR1* pathogenesis-related protein 1. The 2^−ΔΔCt^ values were significantly different between treated and control plants (*p < 0.05; **p < 0.01; Student’s *t* test)

The possible overlap between the pathways activated by prosystemin and by the aphid feeding was investigated analyzing the expression level, following aphid infestation, of genes selected from the microarray study. Leaves were harvested 96 h post infestation, when aphids have established a feeding site. The data indicated that the selected genes (Pin I, Pin II, KPI, LAP, TD, PR1, and WRKY40) were differentially expressed following aphid infestation (Fig. 7). An interesting difference between the response to aphids and to prosystemin was related to PR1 expression, a protein typically produced in plants in the event of a pathogen attack. PR1 was downregulated in the RSYS plant but induced by aphid feeding. The latter is, for instance, consistent with data in *Arabidopsis thaliana* seedlings (Moran and Thompson 2001) and indicated that tomato response to aphids is complex and shares components with both fungal pathogens and herbivores (Coppola et al. 2013; Smith and Boyko 2007; Thompson and Goggin 2006).

## Discussion

The molecular response to wounding and/or herbivory has been investigated in different plant species. Considerable progress has been gained from model systems but different species can also show remarkable differences in relation to JA and systemin signaling (Li et al. 2004; Schmidt and Baldwin 2006). Systemin-mediated defense has represented an important model to elucidate plant signal transduction in response to insect attack in tomato. Following the seminal work of Ryan’s group (Mcgurl et al. 1994), one of the highly expressing transgenic lines has been used in a number of studies to shed light on defense mechanisms against chewing insects and mechanical wounding (Bergey et al. 1999; Chen et al. 2005; Constabel et al. 1995; Dombrowski et al. 1999; Jacinto et al. 1997; Li et al. 2003). Currently, it is widely accepted that the multicomponent response to pests requires an extensive genetic reprogramming and metabolic re-allocation. In absence of a stress, defenses are kept under genetic control in order to be activated only when plants sense the danger. An advantage of the overexpression of prosystemin is to activate JA responses with high specificity without the plausible complicating effects of tissue damage or attacker-derived molecules that target other physiological processes in the host (e.g., water loss, tissue removal, etc.). However, the impact of the systemin peptide on tomato transcriptome, as well as its possible functional outcome in other interactions, has not been systematically addressed. Moreover, large-scale microarray studies on plant response to pests are relatively scarce in tomato and other cultivated Solanaceae (Coppola et al. 2013; Lawrence et al. 2008; Scranton et al. 2013; Strassner et al. 2002; Uppalapati et al. 2005).

The expression analysis, based on two independent single-copy transgenic lines, revealed a higher number of systemin-associated genes than previously reported (Bergey et al. 1999; Ryan 2000), implying a broader effect of this peptide in tomato. The activation of the oxylipin pathway and related defense compounds represents a substantial effect especially considering the fold-change of the gene expression, because genes coding for different proteins that could directly affect pests were the most highly expressed. The inhibitory spectrum of upregulated proteinase inhibitors was wide, as they include members acting against serine, cysteine, and metalloproteases as well as proteins involved in the catabolism of essential amino acids in the insects gut. Despite the metabolic cost of producing a range of proteins with similar biological function, a concerted expression of different PI is essential to provide a long-term influence on phytophagous pests (Lawrence and Koundal 2002; Zhu-Salzman et al. 2008). Nonetheless, prosystemin effects were not restricted to the JA-pathway, since several genes that are dependent on different signaling cascades and pathways were differentially regulated. The expression profile of the transgenic lines presented a degree of overlap with the response programs that are classically associated to pathogen defense or abiotic stress protection. Moreover, different stress-hormone related genes were differentially expressed, providing further evidence that multiple hormone pathways are integrated for defense and wound responses (Robert-Seilaniantz et al. 2011). On the other hand, the transcriptional analysis revealed also a degree of specificity in systemin-induced responses, as subsets of genes related to the JA, SA, ethylene, and brassinosteroid pathways were differentially expressed. As a part of the defense response, the majority of the downregulated genes were connected to carbon metabolism and fixation (Bilgin et al. 2010). As this effect was present in absence of a direct interaction with a biotic stress, out data provided support to the hypothesis that downregulation of photosynthesis is a plant-driven response to the perception of stress, rather than a secondary physiological response to tissue damage and/or water loss. Another relevant feature of prosystemin accumulation was related to phenolic secondary metabolites. Given the wide role of these compounds, only a metabolic analysis would provide sufficient detail to infer a specific effect. Nonetheless, plant phenols and polyphenols are known to increase resistance against fungal pathogens and phytophagous insects. Moreover, if present, an increased lignin biosynthesis and cell wall cross-linking activity would play a broad role in defense against different biotic stressors (Bhuiyan et al. 2009; Nicholson and Hammerschmidt 1992). In addition, taking also into account the previously reported increase in salt-tolerance of prosystemin overexpressing plants (Orsini et al. 2010), the transcriptomics analysis also suggests that prosystemin has the potential to improve both abiotic and biotic stress tolerance of tomato.

Considering the genes differentially expressed, we evaluated the possible functional outcome of prosystemin constitutive overexpression. The bioassay indicated that transgenic plants were more resistant to *Lepidoptera* as expected, but also to aphids and necrotrophic fungi. While the latter can be discussed considering the JA-mediated defenses against necrotrophic pathogens, our data also indicated that other systemin-activated genes, which are not directly linked to the JA-pathway, should contribute to the enhanced performance of the transgenic plants. Genes coding for component of the basal immunity to pathogens (osmotin, miraculin, and extensin) were overexpressed. In tomato, a possible effect of prosystemin on the activation of pathogen defense genes has not yet been investigated but a similar effect was described in potato on different pathogen-associated genes (Bhattacharya et al. 2013). Similarly, although tomato response to aphids and to systemin overproduction is not identical in respect to JA- and SA-dependent genes, our data demonstrated that systemin-response is effective against *M. euphorbiae*. We did not observe differences in the host-acceptance, implying increased plant toxicity. The change in the quality of the host plant is congruent with the inducible production of components directly affecting host feeding. Exogenous JA treatment reduces aphid population growth on a susceptible tomato cultivar (Cooper et al. 2004; Cooper and Goggin 2005) as well as in other species (Gao et al. 2007). More recently, it has been also shown that antisense suppression of the FAD7 gene in tomato, which is associated with enhanced levels of SA, reduces aphid infestations (Avila et al. 2012). Either SA- or JA-dependent acquired resistance in tomato may have a direct negative effect on phloem-feeding insects, as already proposed (Cooper et al. 2004). Considering that the transcriptional analysis indicated that prosystemin overexpression activates genes associated not only to the octadecanoid pathway, further studies are needed to determine how the possible variation in SA, JA, and ET level contributes to the outcome of tomato-biotic stress interaction. Overall, the GO categories of the differentially expressed genes indicated that systemin-dependent plant response is based on the link between the regulation of photosynthesis, hormonal signaling, and production of compounds involved in direct and indirect defense against stress (Kerchev et al. 2012; Wu and Baldwin 2010). The transcriptional response is associated with upregulation of some primary metabolic pathways that would support the accumulation of a number of defense proteins, without a compensatory enhancement of carbon metabolism and photosynthesis. We previously reported that the overexpression of prosystemin associated to a lower CO_2_ assimilation rate and intracellular concentration (Corrado et al. 2011). The enhanced re-routing of metabolism towards primary and secondary compounds involved in defense represents a cost that would explain the slower growth of the plants (Corrado et al. 2011).

Even though the gene ontology of the differentially expressed genes cross biotic-abiotic stress boundaries, these effects are consistent with the proposed role of systemin as enhancer of the herbivore response. Herbivores cause not only tissue removal and cell damage but also increase water loss and susceptibility to opportunistic pathogens.

Besides systemin, a number of plant signaling peptides that activate genes counteracting herbivores or pathogens invasions have been isolated and characterized in different species (Huffaker et al. 2006; Narvaez-Vasquez et al. 2007; Pearce and Ryan 2003; Ren and Lu 2006). In the plant kingdom, defense signaling peptides seem to be involved more often in plant-pathogen interactions (Albert 2013), leading to the proposition that tomato and other members of the Solanoideae subfamily (i.e., potato, nightshade and pepper) may have adapted systemin to strengthen the inducible response to herbivores (Constabel et al. 1998). While genes and signals produced by herbivore or pathogen attack are considered different, the components of the transduction pathways and related gene products may have evolved with some degree of conservation of structure, function or both. It has also been reported that *B. cinerea* enhances prosystemin expression in tomato (El Oirdi et al. 2011). The transcriptomic changes and the broad resistance against biotic stress that we observed suggest that systemin is a component of multiple defense pathways, supporting the proposition that plant-derived peptide elicitors have a more general role in amplifying defense signaling pathways against herbivores, fungal pathogens, or more likely both (Albert 2013; Pearce et al. 2008). Systemin peptide should be then considered a damage-associated molecular patterns (DAMPs) molecule, which is released by stressed cells to dictate an innate immune reaction (Albert 2013; Howe and Jander 2008; Yamaguchi and Huffaker 2011). Moreover, our study implies also that, at least in the absence of a direct interaction with a biotic stressor, a clear dichotomy between pathogen- and herbivore-specific defense pathways does not always exist (Felton and Korth 2000), consistent with the proposed presence in tomato of reciprocal, plant-mediated positive interactions (Stout et al. 1999).

In conclusion, our work provides a detailed overview of the transcriptomic modifications determined by prosystemin overexpression in tomato, leading to a more comprehensive understanding of systemin defense signaling network. The broad effect of prosystemin suggests that a vast array of plant defense processes may be controlled by a relatively reduced number of endogenous molecules. Finally, our data demonstrate that tomato resistance against different biotic stresses can be significantly enhanced by promoting the expression of a single gene that encodes a key component of the inducible defensive systemic signaling system. The effect of prosystemin might well influence different aspects of plant-environment interaction, for instance, not limited to biotic stressors but also affecting beneficial organisms or neighboring plants. Future studies are needed to increase our understanding on how the modulation of an endogenous signal can influence tomato performance in a more complex scenario.

## Electronic supplementary material

Below is the link to the electronic supplementary material.

Supplementary Figure 1 Phenotype of the transgenic lines RSYS 24 and RSYS 32 four weeks after sowing. (JPEG 414 kb)

Supplementary Figure 2 Microarray validation and concordance with the Real Time results. The graph displays the concordance between log_2_-microarray fold change and log_2_-Real Time RQ values on a linear scale (R^2^=0.93). Each dot represent a gene. The genes analyzed were: wound-induced proteinase inhibitor I (InhI); JA-ZIM domain family protein (JA-ZIM); Kunitz-type proteinase inhibitor family protein (Kunitz); lypoxygenase D (LoxD); Mate efflux family protein (Mate); PPO: polyphenoloxidase (PPO); ProSys: prosystemin; Pathogenesis-related protein 1A1 (PT1); Pto-responsive gene 1 (Pto); SAM: S-Adenosyl Methionine (SAM); Subtilisin: subtilisin-like protease (Subtilisin). (JPEG 176 kb)

Supplementary Table S1 (DOCX 19 kb)

Supplementary Table S2 and S3 (XLSX 61 kb)

Supplementary Table S4 and S5 (XLSX 16 kb)

Supplementary Table S6 (DOCX 14 kb)

Supplementary Table S7 (DOCX 14 kb)
