# Supplementary figures and images for: Prosystemin Overexpression in Tomato Enhances Resistance to Different Biotic Stresses by Activating Genes of Multiple Signaling Pathways
Source: Plant Mol Biol Report. 2014 Nov 25;33(5):1270–85. doi: 10.1007/s11105-014-0834-x (PMC4551541; doi:10.1007/s11105-014-0834-x)

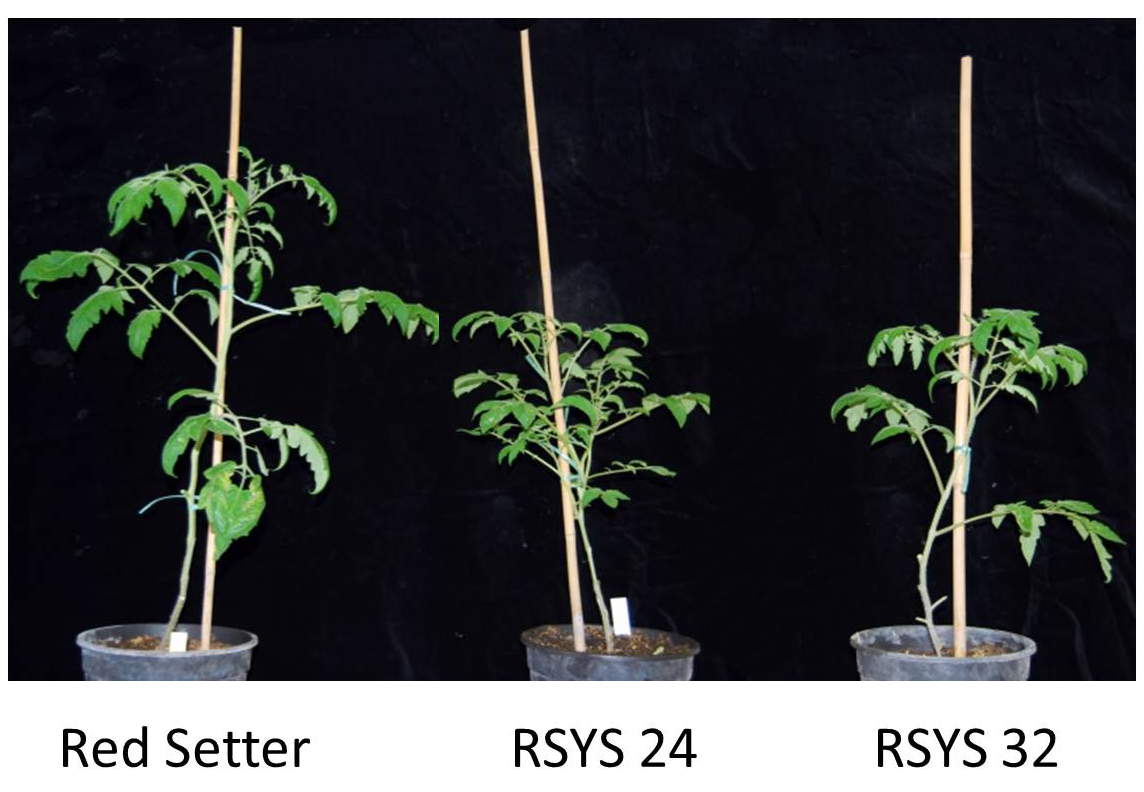

Supplement: Supplementary file 1 — Phenotype of the transgenic lines RSYS 24 and RSYS 32 four weeks after sowing. (JPEG 414 kb) [file 11105_2014_834_Fig8_ESM.jpg]

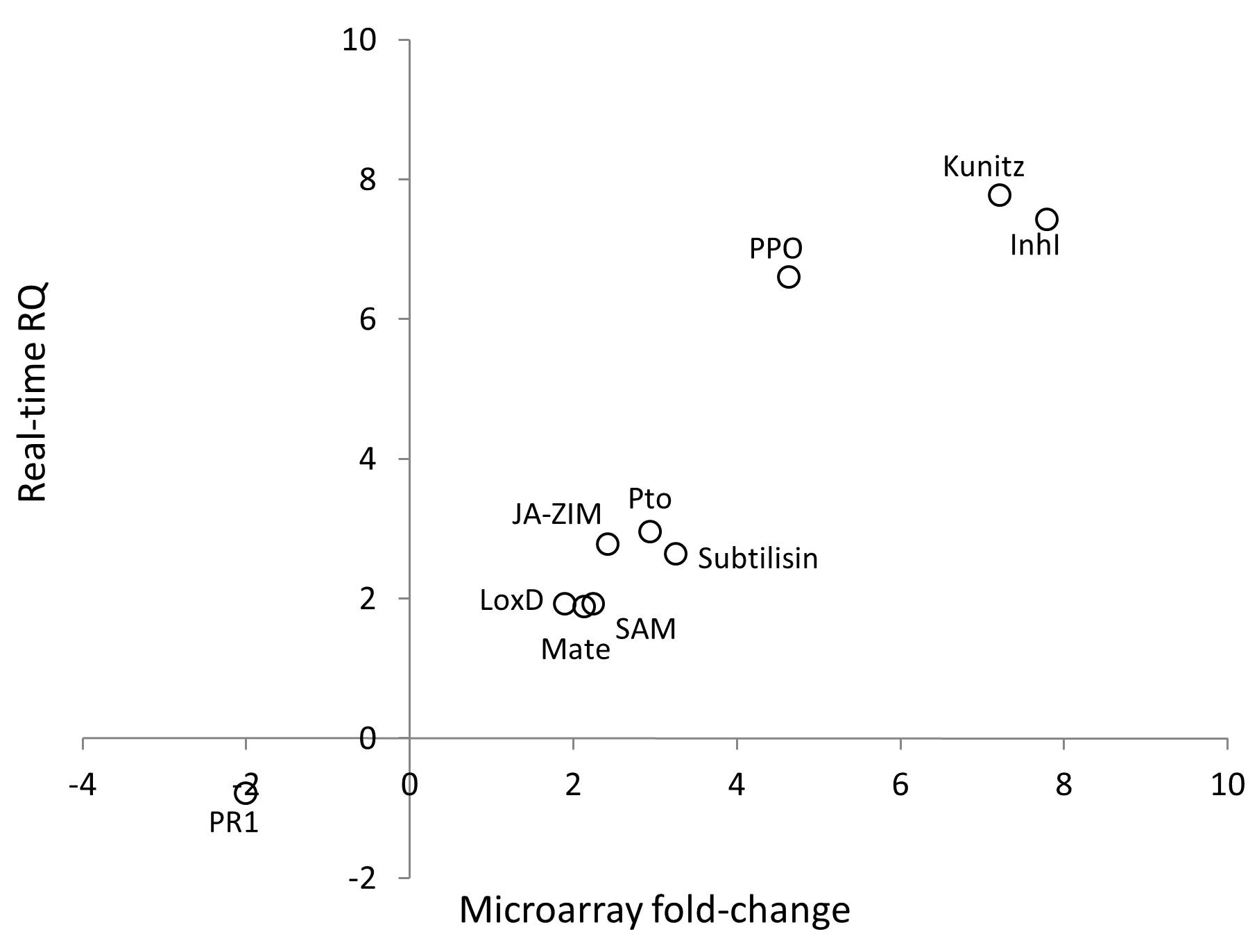

Supplement: Supplementary file 2 — Microarray validation and concordance with the Real Time results. The graph displays the concordance between log2-microarray fold change and log2-Real Time RQ values on a linear scale (R2=0.93). Each dot represent a gene. The genes analyzed were: wound-induced proteinase inhibitor I (InhI); JA-ZIM domain family protein (JA-ZIM); Kunitz-type proteinase inhibitor family protein (Kunitz); lypoxygenase D (LoxD); Mate efflux family protein (Mate); PPO: polyphenoloxidase (PPO); ProSys: prosystemin; Pathogenesis-related protein 1A1 (PT1); Pto-responsive gene 1 (Pto); SAM: S-Adenosyl Methionine (SAM); Subtilisin: subtilisin-like protease (Subtilisin). (JPEG 176 kb) [file 11105_2014_834_Fig9_ESM.jpg]
