# Supplementary material for: Prosystemin Overexpression in Tomato Enhances Resistance to Different Biotic Stresses by Activating Genes of Multiple Signaling Pathways
Source: Plant Mol Biol Report. 2014 Nov 25;33(5):1270–85. doi: 10.1007/s11105-014-0834-x (PMC4551541; doi:10.1007/s11105-014-0834-x)
Supplement: Supplementary file 3 — (DOCX 19 kb) [file 11105_2014_834_MOESM1_ESM.docx]

**Supplementary Table S1. Primer name, sequence, amplicon length, name/gene symbol and accession number of the genes used for the real-time expression analysis**

| **Primer** | **Sequence (5’🡪3’)** | **Amplicon length (bp)** | **Name/Gene synbol** | **Accession Number** |
| --- | --- | --- | --- | --- |
| ARG Fw | AGGTCTCTCTTTCCGCGATGT | 104 | Arginase | AY656837 |
| ARG Rv | CATCAACAGTATCACGCTGCG |  |  |  |
| EF Fw Rt | CTCCATTGGGTCGTTTTGCT | 101 | elongation Factor 1 alpha | X53043 |
| EF Rv Rt | GGTCACCTTGGCACCAGTTG |  |  |  |
| Extensin Fw | CTTGGCCTTTTTTTGGCCAT | 141 | extensin | X55688 |
| Extensin Rv | CGCCATATTCGGCTTCATGT |  |  |  |
| JA-ZIM Fw | AATCGCGAGACGGAATTCACT | 135 | JAZ1 | AK327683 |
| JA-ZIM Rv | GCACCTAATCCCAACCATGCT |  |  |  |
| KPI Fw | TTGTTGGAGACGGAAGGAAGC | 128 | Kunitz-type proteinase inhibitor | X73986 |
| KPI Rv | CGGCAAAATGGACAAAGCAC |  |  |  |
| Lap Fw | ATCTCAGGTTTCCTGGTGGAAGGA | 99 | leucine amino peptidase | U50152 |
| Lap Rv | AGTTGCTATGGCAGAGGCAGAG |  |  |  |
| LoxD Fw | TTCATGGCCGTGGTTGACA | 101 | lipoxigenase D | SLU37840 |
| LoxD Rv | AACAATCTCTGCATCTCCGG |  |  |  |
| Mate Fw | ACCCATCAATGACACCCAAG | 154 | mate efflux protein | BI933305 |
| Mate Rv | GGCATGTGGTATGGGATGTT |  |  |  |
| MCPI Fw | CACAAGACGATTGTTCTGGTGG | 106 | metallo carboxypeptidase | X59282 |
| MCPI Rv | TTGTAATCACACGCCTATGGCC |  |  |  |
| Miraculin Fw | GATACTCCGTACAGGCGTCGAT | 113 | miraculin | SGN-U144553 |
| Miraculin Rv | TGGACAACAGCATCAAGTGGA |  |  |  |
| Osmotin Fw | CCAATATAAACGGTGAATGCCC | 110 | osmotin | AK322591 |
| Osmotin Rv | GACCACATGGACCGTGATTACA |  |  |  |
| PI I Fw | GAAACTCTCATGGCACGAAAAG | 114 | Proteinase inhibitor I | K03290 |
| PI I Rv | CACCAATAAGTTCTGGCCACAT |  |  |  |
| PI II Fw | CCAAAAAGGCCAAATGCTTG | 116 | Proteinase inhibitor II | K03291 |
| PI II Rv | TGTGCAACACGTGGTACATCC |  |  |  |
| PPO Fw | TTTGATAGGCGGAGTTTGCG | 111 | polyphenoloxydase | BI925947 |
| PPO Rv | CCACCAGTTCAGTTATCGCCA |  |  |  |
| PR10 Fw | CATCATGTGACCACGAATGGA | 101 | PR10 | AK329477 |
| PR10 Rv | AACGTGAAGGACAAAACCCAAG |  |  |  |
| PR1a Fw | ATGCAACACTCTGGTGGACCTT | 101 | PR1a | EU589238 |
| PR1a Rv | CCATTGCTTCTCATCAACCCA |  |  |  |
| Pto Fw | TTGCTATGGCTCGTAGAGCAAC | 143 | Pto | TC223474 |
| Pto Rv | CGACGCCGTTTCTTCTTCTT |  |  |  |
| SAM Fw | GCCAAAAATTCTCTGCTTCAGC | 128 | SAM | ES894405 |
| SAM Rv | GAACAACCTAAATCCGCAATGC |  |  |  |
| Subtil Fw | TCAATGGCTGCTCCTCACATT | 102 | subtilisin | TA38526_4081 |
| Subtil Rv | TGCAGTGGTCATCATGGCA |  |  |  |
| Sys Fw | GGGAGGGTGCACTAGAAATA | 161 | prosystemin | M21375 |
| Sys Rv | TTGTCGAAACCGATGATACG |  |  |  |
| TD Fw | TTAGACGCTTTCTCCCCTCGT | 101 | threonine deaminase | M61914 |
| TD Rv | GCTTGAGGAACTTGGAATCCC |  |  |  |
| WRKY40 Fw | GAAAGACAGGCAGCCACTAGGA | 103 | WRKY40 | AK326455 |
| WRKY40 Rv | GCCCATCCCATTTTCACGT |  |  |  |
