# Supplementary material for: Prosystemin Overexpression in Tomato Enhances Resistance to Different Biotic Stresses by Activating Genes of Multiple Signaling Pathways
Source: Plant Mol Biol Report. 2014 Nov 25;33(5):1270–85. doi: 10.1007/s11105-014-0834-x (PMC4551541; doi:10.1007/s11105-014-0834-x)
Supplement: Supplementary file 6 — (DOCX 14 kb) [file 11105_2014_834_MOESM4_ESM.docx]

**Supplementary table 6: KEGG-based association of enzymatic functions of the overexpressed genes in the context of the metabolic pathways in which they participate.**

Only sequences with GO annotations were considered. KEGG-based EC-annotation was performed considering the mapping file available at the Gene Ontology web site (sequence cut-off >2).

| Pathway | | Sequences in Pathway |
| --- | --- | --- |
| Cysteine and methionine metabolism | 13 | |
| Arginine and proline metabolism | 13 | |
| Phenylalanine metabolism | 12 | |
| Phenylpropanoid biosynthesis | 12 | |
| Glutathione metabolism | 10 | |
| Tyrosine metabolism | 10 | |
| Flavonoid biosynthesis | 9 | |
| Isoquinoline alkaloid biosynthesis | 8 | |
| beta-Alanine metabolism | 7 | |
| Stilbenoid, diarylheptanoid and gingerol biosynthesis | 6 | |
| Glycerolipid metabolism | 6 | |
| Aminobenzoate degradation | 6 | |
| Riboflavin metabolism | 5 | |
| Glyoxylate and dicarboxylate metabolism | 5 | |
| alpha-Linolenic acid metabolism | 5 | |
| Drug metabolism - cytochrome P450 | 4 | |
| Metabolism of xenobiotics by cytochrome P450 | 4 | |
| Methane metabolism | 4 | |
| Purine metabolism | 4 | |
| Glycine, serine and threonine metabolism | 4 | |
| Starch and sucrose metabolism | 4 | |
| Drug metabolism - other enzymes | 3 | |
| Tropane, piperidine and pyridine alkaloid biosynthesis | 3 | |
| Ubiquinone and other terpenoid-quinone biosynthesis | 3 | |
| Glycerophospholipid metabolism | 3 | |
| Valine, leucine and isoleucine degradation | 3 | |
| Styrene degradation | 3 | |
| Selenocompound metabolism | 3 | |
| Fatty acid degradation | 3 | |
| Tryptophan metabolism | 3 | |
