# Supplementary material for: Prosystemin Overexpression in Tomato Enhances Resistance to Different Biotic Stresses by Activating Genes of Multiple Signaling Pathways
Source: Plant Mol Biol Report. 2014 Nov 25;33(5):1270–85. doi: 10.1007/s11105-014-0834-x (PMC4551541; doi:10.1007/s11105-014-0834-x)
Supplement: Supplementary file 7 — (DOCX 14 kb) [file 11105_2014_834_MOESM5_ESM.docx]

**Supplementary table 7: KEGG-based association of enzymatic functions of the downregulated genes in the context of the metabolic pathways in which they participate.**

Only sequences with GO annotations were considered. KEGG-based EC-annotation was performed considering the mapping file available at the Gene Ontology web site (sequence cut-off >2).

| Pathway | Sequences in Pathway |
| --- | --- |
| Starch and sucrose metabolism | 8 |
| Amino sugar and nucleotide sugar metabolism | 7 |
| Cysteine and methionine metabolism | 6 |
| Galactose metabolism | 5 |
| Sulfur metabolism | 4 |
| Cyanoamino acid metabolism | 4 |
| Pyruvate metabolism | 4 |
| Carbon fixation in photosynthetic organisms | 4 |
| Fructose and mannose metabolism | 4 |
| Glycosaminoglycan degradation | 3 |
| Glyoxylate and dicarboxylate metabolism | 3 |
| Methane metabolism | 3 |
| Glycosphingolipid biosynthesis - ganglio series | 3 |
| Sphingolipid metabolism | 3 |
| Other glycan degradation | 3 |
